# Comparison of Effects of Light Anesthetics, Diethyl Ether and Carbon Dioxide, on Hypothalamic Paraventricular Nucleus D_1_ and D_2_ Dopamine Receptors- and Glucosensitive Neurons-Induced Food Intake in Fasted Conscious Rats

**DOI:** 10.32598/bcn.9.4.269

**Published:** 2018-07-01

**Authors:** Masoud Shareghi Brojeni, Morteza Salimi, Zahra Mirmohammadsadeghi, Abbas Haghparast, Afsaneh Eliassi

**Affiliations:** 1. Neurophysiology Research Center, Shahid Beheshti University of Medical Sciences, Tehran, Iran.; 2. Department of Physiology, School of Medicine, Shahid Beheshti University of Medical Sciences, Tehran, Iran.; 3. Neuroscience Research Center, Shahid Beheshti University of Medical Sciences, Tehran, Iran.

**Keywords:** Carbon dioxide, Diethyl ether, Paraventricular Nucleus (PVN), Food intake, Dopamine receptors, Glucosensing neurons

## Abstract

**Introduction:**

Carbon Dioxide (CO_2_) and diethyl ether are used as light anesthetics. However, experimental data about their side effects are scarce. In addition, in all our previous works on regulatory mechanisms of hypothalamus during food intake, including the effect of Paraventricular Nucleus (PVN) D_1_ and D_2_ dopamine receptors and glucosensitive neurons, the drug injections were performed under brief diethyl ether anesthesia. In the current study, we tested the hypothesis which postulates that CO_2_ and diethyl ether as light anesthetic agents affect the stimulatory effect of PVN dopamine receptors and glucosensitive neurons in feeding behavior.

**Methods:**

Male Wistar rats were implanted with guide cannula directed to their PVN. Glucose (0.8 μg), SKF38393 (D_1_ agonist, 0.5 μg), quinpirole (D_2_ agonist, 0.3 μg) and saline (0.3 μL) were microinjected into the PVN and food intake was measured over 1 hour.

**Results:**

Our results showed that CO_2_ but not diethyl ether decreased food intake compared to intact animals. The PVN injections of glucose, SKF38393, and quinpirole increased food intake under brief diethyl ether anesthesia. In contrast, the PVN microinjected glucose-induced and dopamine receptor agonists-induced food intake were inhibited under light CO_2_ anesthesia.

**Conclusion:**

Our results suggest that brief exposure to CO_2_ and diethyl ether as light anesthetic agents may affect PVN glucosensing neurons-induced and dopamine receptors-induced food intake in fasted rats.

## Highlights

- Light anesthesia affects food intake.
- Exposure to light anesthetic agents affect the glucosensing and dopaminergic neurons.
- Feeding experimental results may be affected by any experimental approach using light anesthetic agents.

## Plain Language Summary

Carbon dioxide and diethyl ether are used as light anesthetic agents in many experimental approaches. However, experimental data about their side effects are scarce. We found that light CO_2_ but not diethyl ether decreases food intake. Our results demonstrate that hypothalamic neurons may be, at least in part, one of the targets of light anesthetic agents. The current study also suggests that experimental approach using these anesthetics may affect feeding behavior.

## 1. Introduction

In experimental research, inhalation of Carbon Dioxide (CO_2_) or diethyl ether is used as light anesthetic agents. For example, in our previous studies that we considered the role of the Ventromedial Hypothalamus (VMH) (Eliassi, Nazari, & Naghdi, 2009) and paraventricular (Chaleek, Kermani, Eliassi, & Haghparast, 2012; Kermani & Eliassi, 2012) hypothalamic orexin-1 receptors in the regulation of gastric acid secretion, the ventromedial hypothalamus VMH or PVN drug injections were performed under brief diethyl ether anesthesia.

In addition, Zaringhalam, Tekieh, Manaheji, and Akhtari (2013) considered the cellular events during arthritis-induced hyperalgesia under brief CO_2_ anesthesia. However, little is known about the effect of light anesthetic agents on experimental results. Van Herck et al. (1991) demonstrated that rat plasma corticosterone and glucose increased after two minutes exposure to diethyl ether anesthesia. Furthermore, Zardooz et al. (2010) showed that a brief exposure to either diethyl ether or CO_2_ affected the plasma corticosterone, glucose, and insulin levels in fed or fasted rats. These data and others (Tanaka, Nabatame, & Tanifuji, 2005) support that light anesthetic agents affect the experimental data.

Recently, the effects of CO_2_ on insects and plants behavior have been shown. For example, Majeed, Hill and Ignell (2013) demonstrated that the take-off and source contact behavior of Aedes aegypti (female yellow fever mosquitoes) is impeded at elevated background levels of CO_2_ as a result of masking of the stimulus signal. Furthermore, saprophagous insects often use CO_2_ as a cue for finding food (Kojima, 2015) and elevated atmospheric CO_2_ increases fiber fractions of a mammalian herbivore, Microtus ochrogaster (Habeck & Lindroth, 2013).

To control the homeostatic feeding motivation, a number of neurons project to hypothalamic Paraventricular Nucleus (PVN) (Morton, Cummings, Baskin, Barsh, & Schwartz, 2006; Saper et al. 2002; Schwartz et al., 2000). Dopamine is also considered to be the main catecholamine in the brain and serves an important regulatory role in the control of feeding behavior (Szczypka, Rainey, & Palmiter, 2000; Steele et al., 2010). Dopamine signaling is mediated by five receptors, termed D_1_–D_5_ receptors. Administration of D_2_ receptor agonist decreases plasma leptin levels in an obese woman and increases food intake (Kim, Shin, Kim, Lee, & Baik, 2005).

Furthermore, according to Yu and Kim (2012) study, D_4_ receptors in PVN may be a pharmacological target for obesity. Recently, we also reported that D_1_ and D_2_ Dopamine Receptors (DR) and also Glucosensitive Neurons (GSNs) in the hypothalamic Paraventricular Nucleus (PVN) increased food intake in 18 hours food-deprived rats (data is preparing to be submitted). In our experiments, dopamine agonists, antagonists and glucose were injected into the hypothalamic Paraventricular Nucleus under light diethyl ether anesthesia. However, little is known about the effect of brief diethyl ether or CO_2_ anesthesia on experimental food intake results. Therefore, in this study, we evaluated whether inhalation of diethyl ether and CO_2_ as light anesthetic agents is able to affect food intake in conscious rats.

Furthermore, we considered and compared the effect of these two anesthetic agents on PVN D_1_ and D_2_ dopamine receptors-induced and glucose-induced food intake in 18 hours food-deprived rats.

## 2. Methods

### 2.1. Animals

Male Wistar rats, weighing 220–250 g (Neuroscience Research Center, Tehran, Iran) were housed in 12:12 h light:dark cycle at 22°C–24°C. They were deprived of food, but not water, for 18–20 h prior to experiments.

### 2.2. Drugs

Ketamine (Rotex, Levallois-Perret, France) and xylazine (Alfasan, Woerden, The Netherlands) were used to anaesthetize rats. Quinpirole, SKF38393 and glucose were purchased from Sigma (St Louis, MO, USA).

### 2.3. Injection of compounds

Drugs or vehicle were injected in a volume of 0.3 μL into the PVN. The drug injections were performed under brief diethyl ether or CO_2_ anesthesia using a 0.5-μL Hamilton syringe. Animals were exposed to CO_2_ or diethyl ether inhalation for 30 s and obtained full consciousness after 1 minute.

### 2.4. Operation

After anesthetizing by ketamine and xylazine, animals were fitted with a 23-gauge stainless steel cannula. Cannula was inserted into right PVN according to the stereotaxic atlas of Paxinos and Watson (2007) as follows: lateral, 0.4 mm from midline; dorsoventral, 7 mm from skull surface; and anteroposterior, 1.8 mm from the bregma. The injector was extended 1 mm beyond the end of the guide cannula. Experimental trials were performed after 7-day recovery period. For histological examination, the brains were fixed in formalin and 100-μM thick sections were taken and examined with light microscopy.

### 2.5. Measurements of food intake

The weight of food pellets used were measured by a Sartorius scale, TE3135 (Gottingen, Germany), with d=0.001 mg accuracy. Feeding trials normally conducted from Saturday to Wednesday between 9:00 AM and 12:00 PM. On the test day, the fasted rats were transported to the laboratory at least 1 h before the beginning of the feeding trial. After injecting the test compound, the rats were placed in a clear plastic cage and allowed access to a premeasured amount of their regular lab feeding chow. The amount of food and crumbs left in the test cages was measured. Rats received no more than two feeding trials per week. All experiments were approved by the Research and Ethics Committee of Shahid Beheshti University of Medical Sciences (IR.SBMU. PHNS.REC.1396.33).

### 2.6. Statistical analysis

Results are presented as the mean±SEM. The differences between two and more than two groups were evaluated by the Student t test and 1-way ANOVA followed by Tukey HSD test, respectively. P<0.05 was considered significant.

## 3. Results

### 3.1. Influence of light CO_2_ and diethyl ether anesthesia on food intake

**Figure 1.**
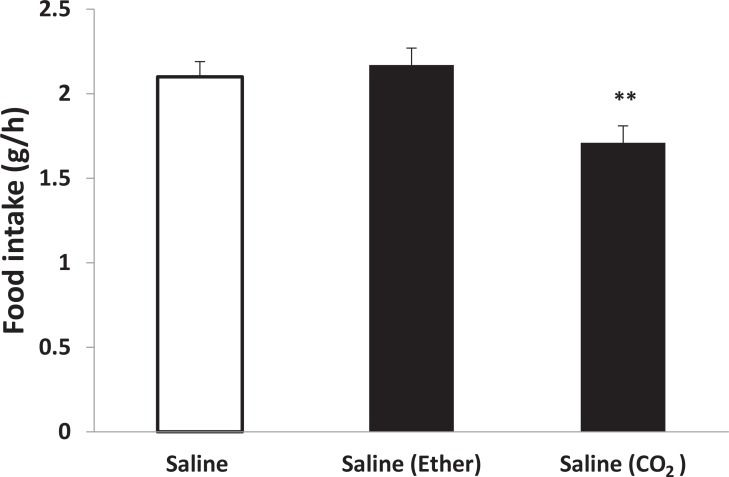
Effects of the light anesthetics on PVN injection of saline in food intake Data are presented as mean±SEM (n=5 per group). ***P<0.001 compared to saline group; **P<0.01 compared with saline group (intact animals).

Inhalation of CO_2_ but not diethyl ether (for 30 s) had a significant effect on food intake. After light CO_2_ anesthesia, food intake decreased significantly (Figure 1). Our results indicate a reduction of approximately 20% with a value of 1.71±0.1 g/h from light CO_2_ test group compared to 2.1±0.1 g/h for the control group (n=5) (P<0.01). Furthermore, Figure 1 demonstrates that light diethyl ether anesthesia has no effect on food intake.

### 3.2. Effects of light CO_2_ and diethyl ether anesthesia on glucose-induced food intake

**Figure 2.**
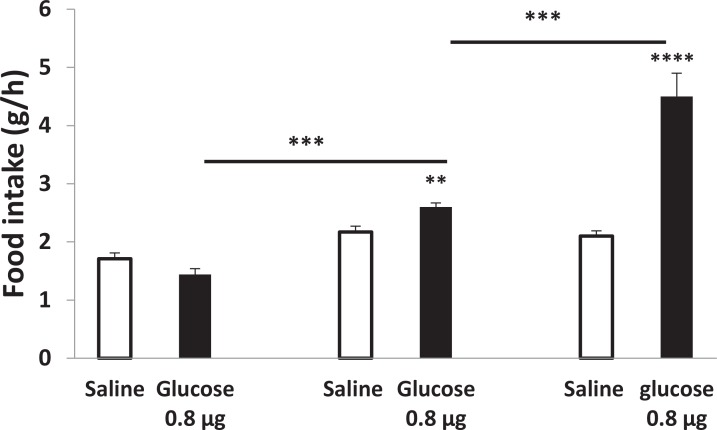
Effects of the light anesthetics on PVN injection of glucose in food intake Data are presented as mean±SEM (n=6 per group). **P<0.01; ***P<0.001; ****P<0.0001 compared to saline group.

Our study showed that the PVN injection of glucose induced dose-dependent increase of gastric acid secretion and glucose 0.8 μg had maximum stimulatory effect (Chaleek, et al, 2012). Acid secretion is a part of feeding behavior. As shown in Figure 2, in light CO_2_ test groups, glucose (0.8 μg) did not affect food intake compared to the control rats (animals received saline without anesthetic agents).

In diethyl ether group, food intake increased from 2.1 g/h in saline group to 2.6±0.08 g/h in glucose 0.8 μg-treated rats (n=5) (P<0.01). In the absence of anesthetic agents, however, the magnitude of glucose-induced food intake was approximately 2-fold more, compared to the control group (Figure 2).

### 3.3. Effects of light CO_2_ and diethyl ether anesthesia on SKF3833 (D_1_ receptor agonist)-induced and quinpirole (D_2_ receptor agonist)-induced food intake

**Figure 3.**
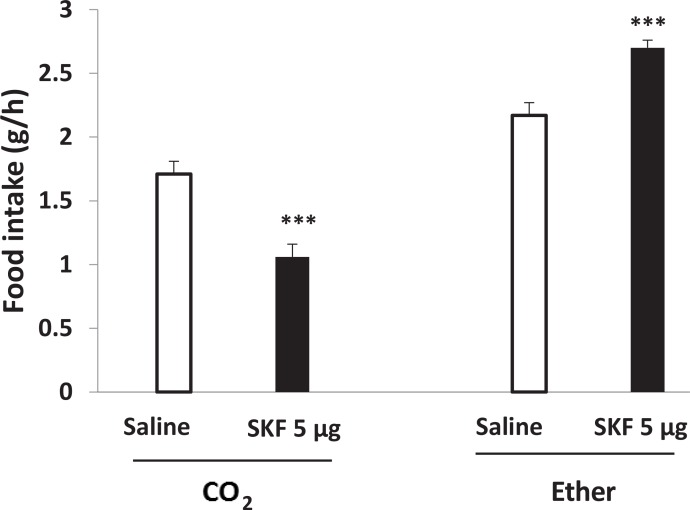
Effects of the light anesthetics on PVN injection of SKF38393 in food intake ***P<0.001 compared to saline groups.

**Figure 4.**
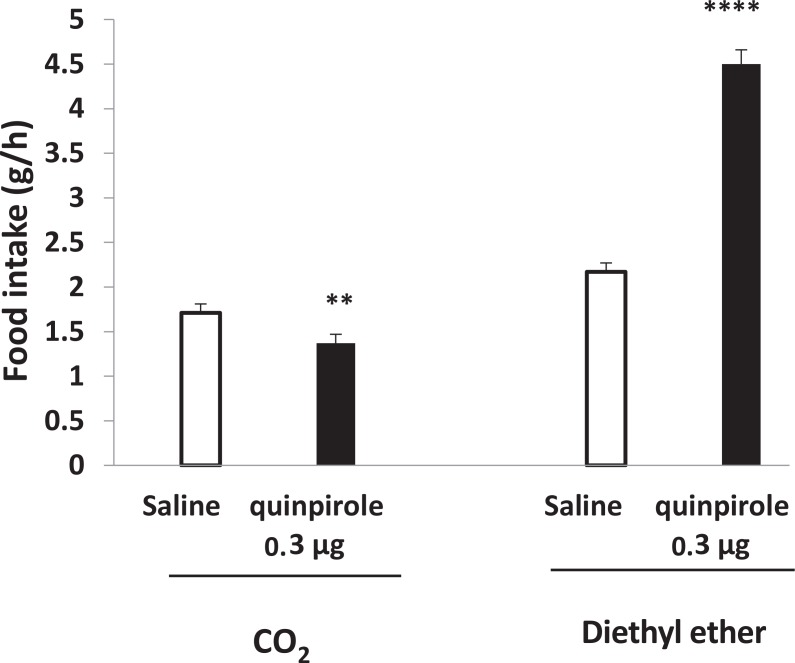
Effects of the light anesthetics on PVN injection of quinpirole in food intake ****P<0.0001; **P<0.01 compared to saline groups.

In our previous study, we showed that PVN-microinjection of SKF38393 and quinpirole increased food in-take in a dose-dependent manner and the maximum effects were observed at doses of 3 and 5 μg, respectively (data is preparing to be submitted). PVN injection of SKF38393 (P<0.001) or quinpirole (P<0.01) decreased food intake after light CO_2_ anesthesia (Figures 3 and 4). Compared to CO_2_ group, light diethyl ether anesthesia had reverse effect on D_1_ and D_2_ receptors-induced food intake. As shown in Figures 3 and 4, PVN microinjection of SKF38393 (5 μg) and quinpirole (0.3 μg) increased food intake compared to saline group (P<0.001 and P<0.0001, respectively).

## 4. Discussion

In this study, we demonstrated that brief inhalation of CO_2_, but not diethyl ether, as light anesthetic agents, decrease food intake compared to saline-treated rats. Furthermore, in the current study, we found that D_1_ and D_2_ dopamine receptors-induced food intake decreases under light CO_2_ anesthesia. However, despite the negative effect of CO_2_ on D_1_ and D_2_-induced food intake, we observed D_1_ and D_2_ agonist increased feeding behavior under brief diethyl ether anesthesia. Our result has also shown that glucose-stimulated food intake has remained at high level under light diethyl ether anesthesia similar to intact animals (without light anesthetic agents). However, this effect was not observed in CO_2_ group with the same drug condition.

The hypothalamic Paraventricular Nucleus (PVN) receives a number of central pathways to control the eating behavior (Blouet & Schwartz, 2010; Morton et al., 2006; Schwartz et al., 2000). These studies have demonstrated that the neuropeptides and neurotransmitters are involved in these phenomena. For example, anorectic agent induces its effects through the cerebral release of dopamine, and the consequent activation of D_1_-like and D_2_-like receptors (Leibowitz, 1975; Chen et al., 2001; Kuo, 2002; Kuo, 2003), decreasing the level of hypothalamic Neuropeptide Y (NPY) (Hsie, Yang, & Kuo, 2005; Kuo, 2005). Furthermore, we have shown that PVN-microinjected SKF38393 (a dopamine D_1_ agonist) and quinpirole (a dopamine D_2_ agonist) increased food intake at doses more than 0.07 μg. These effects were inhibited by D_1_ and D_2_ dopamine receptor antagonists, SCH23390 and sulpiride, respectively (data are preparing to be submitted).

Within the hypothalamus, glucosensitive neurons are found in the arcuate and paraventricular nuclei (Silver & Erecinska, 1998). Our results showed that the PVN-microinjected glucose increased gastric acid secretion at doses of 350–750 nM in 18–24 h fasted conscious rats (Chaleek, et al, 2012). Gastric acid secretion is a part of feeding behavior. Therefore, we suggest that the PVN-glucose sensing neurons might be involved in central regulatory mechanism of acid secretion and the control of energy homeostasis.

All our experiments were done under brief diethyl ether anesthesia. Although it is well established that PVN D_1_ and D_2_ dopamine receptors and glucosensing neurons are involved in regulatory mechanisms of feeding behavior, the specific effects of inhalation of light anesthetic agents including CO_2_ and diethyl ether during experimental approaches have remained unexplored. Our results show that glucose and D_1_ and D_2_ agonists increase food intake under brief diethyl ether anesthesia whereas light CO_2_ inhalation inhibits the effect of glucose and changes the stimulatory effects of D_1_ and D_2_ agonists to inhibitory effects in feeding behavior. At the present time, we do not know the exact CO_2_ and diethyl ether mechanisms on food intake.

Probably the food intake decreases under brief anesthesia as a result of masking of the stimulus signal. For example, PVN and lateral hypothalamus received NPY-containing neuron projections from arcuate nucleus. It has been shown that NPY increases food intake by activating NPY1 and NPY5 receptors within the hypothalamus (Levens & Della-Zuana, 2003; Mashiko et al., 2006). Furthermore, our previous studies indicate that the orexin-A-induced gastric acid secretion in PVN (Chaleek, et al, 2012) is blocked by Intracerebroventricular (ICV) administration of NPY1- and NPY5-receptor antagonists (Kermani & Eliassi, 2012).

Gastric acid secretion is a part of feeding behavior. Meguid et al. (2000) demonstrated that afferent information from the autonomic nervous system affects gastrointestinal mediators, and circulatory concentrations of nutrients and hormones are transmitted to the presynaptic monoaminergic system of the hypothalamus. These presynaptic afferent neurons influence postsynaptic cells by releasing dopamine. According to their model, post-synaptic neurons may express both D_1_ and D_2_ receptors which are involved in food intake by activation of stimulatory and inhibitory food intake neuropeptides, including NPY. Therefore, we suggest that masking of the first or second order hypothalamic neurons in response to the light anesthetic agents may be one mechanism by which food intake decreases.

In conclusion, the present study demonstrates that light CO_2_ but not diethyl ether anesthetics decreases food in-take. Our results suggest that dopamine receptors and glucosensing neurons in PVN may be, at least in part, one of targets of light anesthetic agents. Whether these effects result from masking of inhibitory or stimulatory neurons which originate in the PVN or is mediated by fibers of passage is yet to be determined. The current study also suggests that feeding experimental results may be affected by any experimental approach using these anesthetics

## Ethical Considerations

### Compliance with ethical guidelines

All experiments were approved by the Research and Ethics Committee of Shahid Beheshti University of Medical Sciences (IR.SBMU.PHNS.REC.1396.33).
